# Moral Injury in Former Child Soldiers in Liberia

**DOI:** 10.1007/s40653-021-00414-5

**Published:** 2021-10-23

**Authors:** Pui-Hang Wong

**Affiliations:** 1 Maastricht Graduate School of Governance, Maastricht University, Boschstraat 24, 6211 AX Maastricht, Netherlands; 2 United Nations University MERIT, Maastricht, Netherlands

**Keywords:** Moral injury, Mental health, Child soldiers, Ex-combatants, Reintegration, DDR, Liberia

## Abstract

Moral injury (MI) is a form of traumatic stress induced by perpetrating actions that transgress a person’s beliefs and values. Existing research on MI has been mostly confined to military veterans, however there is reason to believe that the risk of MI among child soldiers is higher due to their age and history of abduction. This study examined the risk of MI in former child soldiers in Liberia and tested whether age and history of abduction moderate the relationship between perpetrating violence and MI based on a sample of 459 former child soldiers. Results from regression analysis confirmed that perpetrators had a higher risk of MI. However, while younger perpetrators were more vulnerable to MI, abduction history had no statistically significant moderation effect on the risk of MI. Further analysis also revealed that the moderation effects are primarily on anxiety, avoidance and negative feelings but not re-experiencing. These findings suggest that new tests and treatment models may be required for future disarmament, demobilization, rehabilitation and reintegration (DDRR) policy.

## Introduction

Although the Liberian Civil War has been over for many years, reintegration remains a challenge for many former child soldiers who are in their prime ages in Liberia today. Many of them remain stigmatized and psychologically burdened because of their past (McMullin, 2021; Veale et al., 2017). As thousands of child soldiers have been recruited in the wars in Liberia and other countries (US Department of State, 2021; Wessells, 2006), rehabilitation and reintegration issues associated with this vulnerable group are imminent policy challenges for the government in many post-conflict societies.

Psychological rehabilitation of former child soldiers usually centers on the assessment, prevention and treatment of post-traumatic stress disorder (PTSD) (e.g., Betancourt et al., 2020; Derluyn et al., 2004; Kizilhan & Noll-Hussong, 2018; Murphy et al., 2017). Concurrently, recent literature on psychological trauma is debating to what extent PTSD is (clinically) distinct from moral injury (Barnes et al., 2019; Griffin et al., 2019; Litz et al., 2009). Moral injury (MI) is a form of traumatic stress induced by exposure to psychologically injurious events such as perpetrating actions that transgress a person’s core beliefs and values (Griffin et al., 2019; Litz et al., 2009). While MI may include symptoms of PTSD (Braitman et al., 2018; Litz et al., 2009), it could be present without PTSD and could be less responsive to some psychotherapies for PTSD (Evans et al., 2021; Gray et al., 2017). As such, there is a need to examine the reintegration and rehabilitation of former child soldiers through the lens of MI, as the MI perspective may lead to new preventive approaches and treatment models that help child soldiers to better recover and integrate back into civilian life.

Thus far, studies of MI on former combatants have focused on military veterans (Frankfurt & Frazier, 2016) and extended to other high-risk groups such as medical professionals and social workers; MI in former child soldiers, however, remains under-studied. As children’s and adolescents’ moral agency is still in development, they may experience difficulties integrating their wrongdoing with their moral selves and are affected by their wartime experience differently than adults (Wainryb, 2011). While they still understand that hurting other people is wrong, they could have a different understanding and interpretations on the implications of their behaviors in a usually complex moral scenario (Habermas & de Silveira, 2008; Komolova et al., 2017; Pasupathi & Wainryb, 2010; Wainryb, 2011). In a qualitative analysis based on the narrative accounts from 53 former child soldiers in Colombia, Wainryb (2011) describes three types of moral agency constructions, namely numbing, constrained and split agency, and essentialized construction. These different construction types could be associated with distinct mental processes and emotional responses, such as numbing, fear, self-condemnation, puzzlement, shame, regret, guilt and the mix of them (Chaplo et al., 2019; Wainryb, 2011). Furthermore, when compared with the adult combatants, they could be at greater risk of developing trauma and other impairments in their basic regulatory processes in the brain, which could further influence their neurobiological, cognitive, emotional and social development (Kidwell & Kerig, 2021; Masten & Narayan, 2012; Perry et al., 1995; Wainryb, 2011). Given the developmental implications of MI and their relationship with moral agency in children and adolescents, it is important to consider child soldiers’ war-related adversity beyond the lens of PTSD.

In addition to developmental factors, many other factors have found to be key determinants or moderators of children’s psychological responses to extreme adversity. These factors include personal dispositions, adaptive capacities, previous exposure to trauma, trauma severity, their roles in the injurious events, choice of coping strategies, attachment relationships with caregivers, gender, the socioeconomic status of the former combatants, the existence and kind of community supports and sociocultural context (Bonanno et al., 2015; Haight et al., 2016; Harnisch & Montgomery, 2017; Klasen et al., 2010; Masten & Narayan, 2012; Osofsky, 1999). In the same vein, this study explores two variables which may moderate the effects of transgressions on the risk of MI. The first variable is age at first atrocity. The age that atrocities are committed is usually lower for child soldiers than recruited military professionals. Since MI is induced by transgressions that violate a person’s moral values, the development of MI is likely to depend on the age when perpetrations occur. While children and youths might be psychologically more adaptive than adults (Beirens, 2008; Boyden, 2003), some studies suggest that former child soldiers can be psychologically more susceptible to traumatic experience than adult ex-combatants (Hermenau et al., 2013; Wainryb, 2011). Since children’s views on authority, justice and transgression may progress through stages (Boyden, 2003; Kohlberg et al., 1983; Masten & Narayan, 2012; Wainryb, 2011), and the development of MI hinges partly on stages of development, personal beliefs, values and how these are understood (Currier et al., 2015; Kidwell & Kerig, 2021), child soldiers’ psychological resilience in terms of vulnerability, susceptibility to external influence, self-perceived culpability, and capacity to adjustment and re-alignment may make them more likely to develop MI (Haight et al., 2016; Wainryb, 2011). Accordingly, the age that a former child soldier joined an armed group is likely to affect the risk of MI. This study will test this hypothesis by examining whether the age that a young ex-combatant joins a faction moderates the relationship between perpetrating violent acts and the risk of MI.

The second variable is whether a child soldier joined a faction involuntarily due to abduction. The reasons for participating in war often vary, from private gains to revenge, to defending their communities, families and honor, among others (Humphreys & Weinstein, 2008; Souleimanov & Aliyev, 2015; Wessells, 2006). However, child soldiers often participate in a war involuntarily after abduction (Thompson, 1999; Wessells, 2006). As people who join forces voluntarily might be easier to create meaning of their actions and is subject to a lower risk of MI (Currier et al., 2015), the voluntary basis of the joining decision may affect the risk of MI. In an early study, Punamäki (1996) finds that a strong ideological commitment can reconcile moral dilemmas among young Jewish children in Israel. In another study, Brett and Specht (2004) also show that former combatants who joined a force voluntarily were highly conscious of their acts and psychologically resilient. However, abduction and forced conscription are common in wars (Podder, 2011; Vindevogel et al., 2011). Armed groups frequently use fear, humiliation, brutality and psychological manipulations to enforce the obedience of the abducted children (Wessells, 1997). Since this learned inability to refuse can induce emotional conflicts, abduction may not eliminate but induce the feelings of shame and guilt, which may lead to MI (Frankfurt & Frazier, 2016; Thomason, 2016). This study will test this hypothesis by examining whether forced recruitment via abduction moderates the relationship between committed atrocities and the risk of MI.

In short, the age and the circumstances that potential moral injurious events (PMIEs) occurred may affect the risk of MI. This study aims to investigate whether these two variables are associated with the risk of MI among former child soldiers based on secondary survey data from a reintegration program administered in Liberia. Due to the paucity of data on former child soldiers, this paper is one of the first quantitative studies that investigates the moderating role of abduction and age on the relationship between perpetration and MI in former child soldiers. Answers to the questions are important, as a better understanding of the potential impact of the two contextual factors may contribute to the future development of the assessment questionnaires and psychotherapy models for child soldiers in disarmament, demobilization, rehabilitation and reintegration (DDRR).

## Method and Data

The data for the analysis are derived from the baseline Innovations for Poverty Action (IPA) Survey about a reintegration program in Liberia (Blattman & Annan, 2016). Conducted in 2009, the reintegration program provided agricultural training, capital inputs and counseling to ex-combatants in Liberia. Individuals were recruited from 138 communities in Central Bong County and eastern Sinoe County, both of which were identified by the United Nations Mission in Liberia (UNMIL) as economically vulnerable regions (McMullin, 2013). The final sample includes 459 ex-combatants (450 males and 9 females). Among them, 380 individuals reported a history of abduction.

### Measures

Although various measures of MI have been proposed in the literature, there is no agreed measurement standard (Griffin et al., 2019). Nevertheless, some defining characteristics of MI have been proposed: shame, reexperiencing, anxiety, withdrawal, avoidance and numbing (Braitman et al., 2018; Frankfurt & Frazier, 2016; Litz et al., 2009). Since the survey contains some but not all items in validated measures of MI, an additive indicator based on some of the above characteristics was used in this analysis. The indicator consists of 20 items clustered in three subscales: (i) reexperiencing, (ii) avoidance and negative feelings, and (iii) anxiety. The items in the first two subscales roughly correspond to Criteria B and C of PTSD in the fourth edition of the Diagnostic and Statistical Manual of Mental Disorders (DSM-IV; Weathers et al., 1993). All 20 items, ranging from 0 (never) to 3 (often), refer to the experiences of the respondents four weeks prior to the interviews. Further details of the aggregated indicator, including the internal consistency of the items, are reported in Table 1.

**Table 1:** Items Included in the Moral Injury Indicator

Cluster	Item
Reexperiencing (alpha = 0.73)	Intrusive recollections of past events
	Recurrent distressing dreams about the past events
	Feeling as if the events recurring
	Physiological reactivity when recalling the experience
Avoidance and negative feelings (alpha = 0.77)	Avoiding places, people and conversations that arouse recollection
	Diminished interest in activities with friends
	Not sharing feelings and ideas with other people
	Not wanting to plan or losing hope for the future
	Feeling of distress because of past experience
	Feeling bad about themselves
	Feeling not important to anybody
	Inability to feel satisfied
	Feeling sad
Anxiety (alpha = 0.72)	Trouble hearing people when recalling the experience
	Uncontrolled vexation
	Being scared for no reason or always checking around
	Jerking when there is sudden noise
	Quick to react against others
	Feeling of heart burning
	Feeling frustrated

The main independent variable is *Violent Acts*. It indicates how many violent acts that the respondents committed when they were in an armed group. It is a 4-point ordinal variable, ranging from 0 (none) to 3 (plenty). The variable *Abducted* is a binary variable. It indicates whether the respondents were abducted into a faction. *Joining Age* is the (biological) age at which the respondents were first joined a faction. *Time in faction* measures the number of months that the respondents stayed in warring factions.

Five variables are used to capture the social relations of the respondents to their families, friends and neighbors. *Neighbor (faction)* and *Neighbor (now)* are ordinal variables. They measure the extents to which the respondents experienced problems on gaining acceptance from neighbors shortly after they left the factions and at the time of interviews. Both variables range from 0 (none) to 3 (plenty). *Friend* is a binary variable, coded as one when other ex-combatants constituted more than half of the respondents’ primary groups. *Commander* is a count measure, which is the sum of three binary variables: (i) whether the respondents had maintained a close relationship with their ex-commanders, (ii) whether the respondents had regularly reported to their ex-commanders, and (iii) whether the respondents obtained support (e.g., jobs) from their ex-commanders. *Family* is a composite indicator, which is the sum of six 4-point variables, ranging from 0 (never) to 3 (often): how often the respondents attended family meetings, how much other family members cared about them, supported them, helped them when needed and how often they had fights or other troubles with the respondents (scale reversed). A higher score means a better family relation. Finally, demographic and socioeconomic characteristics are captured by four variables: *age*, *gender* (female = 1), years of *education* and *poverty*. *Poverty* is a binary variable, which equals to one if the respondents earned less than US$1.25 a day in 2009.

To account for the effects of traumatic events, which can also induce MI, the following life-threatening and traumatic experiences are controlled for: shooting at the respondents (yes = 1), attacking the respondents with cutlass or weapons (yes = 1), serious beating (yes = 1), forced sex (yes = 1), battling on the frontline or witnessing battles (yes = 1), having a family member killed during the war (yes = 1), and receiving serious injuries in a battle or an attack (yes = 1). They refer to the experience of the respondents when they were in a warring faction. They take the value 1 if the respondents indicated that they experienced the event. Descriptive statistics of the variables are summarized in Table 2.

**Table 2:** Descriptive Statistics

Variable	N	Mean	Std Dev	Min	Max
Violent acts	459	0.763	1.054	0	3
Abducted	459	0.828	0.378	0	1
Age of joining	459	19.074	6.618	4	50
Age	459	29.632	7.127	18	57
Time in faction (months)	459	30.978	27.805	1	169
Friends	459	0.368	0.483	0	1
Commander	459	0.155	0.190	0	3
Family	459	13.514	3.135	2	18
Neighbor (faction)	459	0.407	0.972	0	3
Neighbor (now)	459	0.146	0.593	0	3
Poverty	459	0.625	0.485	0	1
Female	459	0.020	0.139	0	1
Education (years)	459	5.765	3.635	0	14
Reexperiencing	459	0	1	–1.512	2.424
Avoidance	459	0	1	–1.787	2.905
Anxiety	459	0	1	–1.467	3.255
Moral injury index	459	20.344	10.997	0	56
Shot	459	0.603	0.49	0	1
Cutlass	459	0.695	0.461	0	1
Beat	459	0.601	0.490	0	1
Forced sex	459	0.037	0.189	0	1
Battles	459	0.867	0.34	0	1
Family killed	459	0.922	0.269	0	1
Battle injury	459	0.418	0.494	0	1

### Model

The moderation effects of the joining age and abduction history are tested with a saturated three-way interaction model. The model includes the terms *Violent Acts*, *Age of Joining* and *Abducted*, their interactions and a set of control variables. Parameters are estimated with the ordinary least squares (OLS) method. Robust standard errors are used to adjust for potential heteroskedasticity.

## Results

The estimated effect of having committed violent acts on the MI indicator is reported in Table 3. Model 1 is the baseline model without any interaction terms. Respondents who committed only few violent acts, on average, have a MI score 2.89 units higher (*p* = 0.009) than people who did not commit any. The effect for having committed some violent acts was 4.92 (*p* = 0.015*)* and the effect was 7.66 (*p* = 0.000*)* for those who committed plenty of violent attacks. The positive association is consistent with recent studies on combat veterans (e.g., Nichter et al., 2020; Williamson et al., 2020; Zerach & Levi‐Belz, 2018).

**Table 3:** Estimated Effects of Perpetration, Abduction and Joining Age on MI Risk

	(1)	(2)	(3)	(4)	(5)
Dependent variables	MI	MI	Reexperiencing	Avoidance	Anxiety
Act (few)	2.894***	20.780***	1.300**	1.660***	1.883***
	(1.096)	(6.671)	(0.590)	(0.633)	(0.673)
Act (some)	4.923**	26.367***	0.838*	3.307***	1.462**
	(2.023)	(8.283)	(0.451)	(1.169)	(0.575)
Act (plenty)	7.658***	36.956***	1.707*	3.255***	3.403***
	(1.605)	(7.593)	(0.931)	(0.666)	(0.918)
Abducted (yes)	5.320***	12.797***	0.563	1.151***	1.169***
	(1.237)	(4.118)	(0.382)	(0.394)	(0.413)
Joining (age)	–0.097	0.229	–0.004	0.014	0.039*
	(0.130)	(0.203)	(0.018)	(0.019)	(0.021)
Act (few) x Abducted		–18.043**	–1.076	–1.207*	–1.968***
		(7.657)	(0.687)	(0.726)	(0.760)
Act (some) x Abducted		–20.193**	–0.165	–3.224**	–0.594
		(10.237)	(0.678)	(1.307)	(0.832)
Act (plenty) x Abducted		–23.958**	–0.517	–2.352***	–2.326**
		(9.765)	(1.049)	(0.847)	(1.107)
Act (few) x Joining		–0.566*	–0.042	–0.037	–0.057*
		(0.315)	(0.029)	(0.032)	(0.029)
Act (some) x Joining		–1.120***	–0.024	–0.137***	–0.075***
		(0.342)	(0.018)	(0.047)	(0.026)
Act (plenty) x Joining		–1.333***	–0.061	–0.109***	–0.134***
		(0.308)	(0.040)	(0.027)	(0.035)
Abducted x Joining		–0.243	–0.004	–0.021	–0.028
		(0.208)	(0.018)	(0.020)	(0.021)
Act (few) x Abducted x Joining		0.494	0.037	0.017	0.069**
		(0.370)	(0.034)	(0.037)	(0.034)
Act (some) x Abducted x Joining		1.055**	0.019	0.153***	0.043
		(0.440)	(0.029)	(0.055)	(0.039)
Act (plenty) x Abducted x Joining		0.998**	0.024	0.085**	0.111**
		(0.450)	(0.047)	(0.038)	(0.049)
Age	0.043	0.025	0.007	0.010	–0.012
	(0.119)	(0.114)	(0.011)	(0.011)	(0.011)
Time in faction (months)	0.005	0.004	0.000	–0.000	0.001
	(0.019)	(0.019)	(0.002)	(0.002)	(0.002)
Friends	2.311**	2.280**	0.228**	0.194**	0.131
	(0.967)	(0.967)	(0.092)	(0.089)	(0.094)
Commander	1.123	1.209	0.084	0.130	0.062
	(0.924)	(0.938)	(0.084)	(0.084)	(0.100)
Family	–0.519***	–0.535***	–0.030**	-0.060***	–0.029**
	(0.148)	(0.142)	(0.014)	(0.013)	(0.014)
Neighbor (faction)	1.104***	0.943**	0.093**	0.040	0.105**
	(0.418)	(0.436)	(0.044)	(0.043)	(0.048)
Neighbor (now)	0.182	0.478	0.057	–0.022	0.099
	(0.757)	(0.714)	(0.066)	(0.070)	(0.081)
Poverty	–0.356	–0.604	0.120	–0.131	–0.065
	(0.957)	(0.955)	(0.094)	(0.089)	(0.088)
Female	2.675	3.016	0.269	0.147	0.334
	(3.193)	(3.059)	(0.282)	(0.269)	(0.260)
Education (years)	–0.155	–0.124	–0.001	–0.009	–0.017
	(0.131)	(0.131)	(0.013)	(0.012)	(0.012)
Shot	2.452**	2.670**	0.205**	0.243**	0.177*
	(1.069)	(1.065)	(0.102)	(0.099)	(0.100)
Cutlass	1.570	1.551	0.152	0.122	0.103
	(1.203)	(1.196)	(0.112)	(0.110)	(0.110)
Beat	0.582	0.430	0.076	-0.052	0.113
	(0.955)	(0.967)	(0.098)	(0.091)	(0.092)
Forced sex	7.349***	7.003**	0.324	0.733***	0.499**
	(2.711)	(2.756)	(0.229)	(0.261)	(0.251)
Battles	–0.879	–0.689	–0.206	0.062	–0.092
	(1.589)	(1.583)	(0.154)	(0.137)	(0.145)
Family killed	–1.164	–1.301	–0.138	–0.125	–0.051
	(1.668)	(1.610)	(0.149)	(0.162)	(0.136)
Battle injury	–0.264	–0.271	0.058	–0.078	–0.007
	(1.007)	(0.993)	(0.095)	(0.094)	(0.099)
Constant	19.665	11.781*	–0.521	–0.633	–0.851*
	(3.610)	(4.376)	(0.411)	(0.413)	(0.453)
Observations	459	459	459	459	459
R-squared	0.287	0.319	0.228	0.285	0.257

Robust standard errors in parentheses; * p < 0.1; ** p < 0.05; *** p < 0.01

Social relations also play an important role in the development of MI. Having mainly ex-fighters in one’s primary group is correlated with a higher MI score (2.31 points; *p* = 0.017), though connections with ex-commanders do not have a statistically significant impact on MI score. Neighbors’ acceptance at the time when the respondents just left the faction shows a positive association with the MI score, but as time passes, the effect of neighbors’ acceptance appeared to wane as neighborly acceptance at time of interview has no statistically significant association with MI score. The result suggests that family and other social supports seem to be important protective factors, as they are negatively related to the impact of potential moral injurious events (PMIEs) (Betancourt et al., 2010; Levi-Belz et al., 2020).

**Fig. 1:**
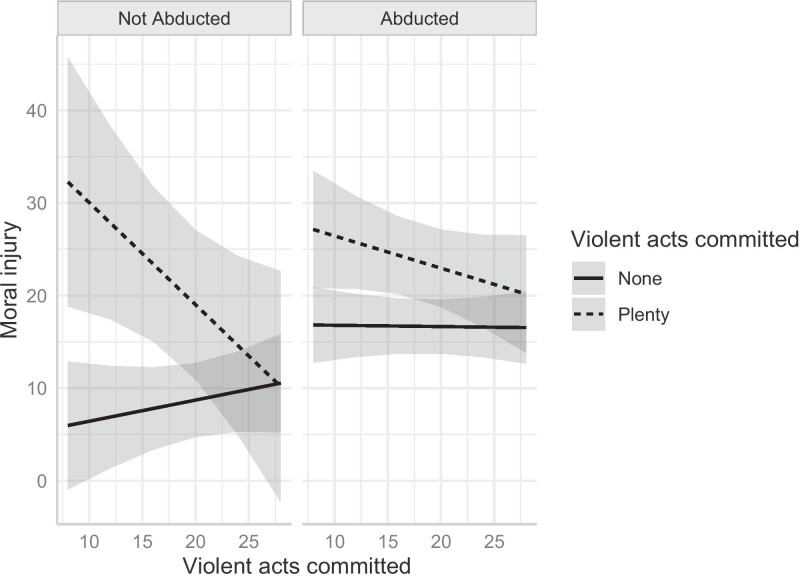
Moderation Effects of Age and Abduction History on MI

The association of past wrongdoings with MI varies depending on the age when a person joined an armed group and whether the person was abducted. Estimates from the model with interaction terms are shown in column 2. The coefficients of most of the interaction terms are statistically significant at the five percent significance level. The moderation effects of atrocities committed on MI scores are shown in Fig. 1. The average marginal effect of perpetrating atrocities is captured by the distance between the solid line and the dashed line. If the 95 percent confidence intervals do not overlap, it means that the marginal effects of perpetrating atrocities are statistically significant.

The correlation between MI and violent acts for the non-abductees is shown in Fig. 1 (left-hand panel). Because the dashed line for ex-fighters who committed plenty of atrocities is higher than the solid line for those who did not commit any, perpetration of atrocities is positively associated with symptoms consistent with MI. For abductees (right-hand panel of Fig. 1), the marginal effects are (slightly) significant for adolescents who joined a faction at the ages between 10 and 17.

The moderation effect of joining age is captured by the distance between the solid and the dash lines of Fig. 1. Whether a younger person joined a group voluntarily or not, the marginal effect of perpetrating atrocities decreases as joining age increases, suggesting that the association between perpetration and the risk of MI is greater for younger people. In other words, younger perpetrators may be more susceptible to MI than their older counterparts. This is contrary to the hypothesis that younger people are more resilient to (certain) trauma stresses and more adaptive to the contextual environment (Bonanno & Mancini, 2012; Tang et al., 2017). For non-perpetrators, joining a faction at a younger age appears to be associated with a lower risk of developing MI, though the shallow slope of the line indicates a mild effect. The upward sloping line on the left-hand panel is steeper for non-abductees, suggesting that younger volunteers (who did not perpetrate violent acts) might be more resilient to MI, a pattern consistent with the hypothesis of meaning-making (Currier et al., 2015; Keller et al., 2020). In short, the findings are consistent with the idea that child soldiers’ minds seem to be adaptive to the external environment but are vulnerable to the stress induced by their wrongdoings.

The moderation effects of the variable *abducted* is captured by the relative size of the gaps between abductees (right-hand panel) and voluntary recruits (left-hand panel). For those who joined a faction as a teenager, the size of the gap is smaller for abductees than for non-abductees. Note that the confidence intervals of the dashed lines are on a similar level. This suggests that, for perpetrators, the history of abduction does not appear to moderate the effect of their wrongdoings. In other words, the relative size of the gaps between the dashed and solid line is mainly driven by the lower position of the solid line in the left-hand panel. A statistically significant difference exists between the solid lines for teenagers. The lower position of the solid line on the left graph means that non-abductees on average have a lower risk of MI than abductees, provided that they did not perpetrate violent acts. Abductees appear to have a higher risk of developing MI, possibly because of their abduction experience. The confidence intervals of the solid lines do not overlap until the joining age reaches approximately 22; this pattern can be observed in the steeper upward slope of the solid line in the left-hand panel, a feature unique to those who did not perpetrate violent acts. The diminishing gap between the lines for both abductees and non-abductees implies that the lower risk associated with volunteering becomes negligible as an individual joining a group as an adult. In seminal work by Punamäki (1996) and Brett and Specht (2004) it is postulated that voluntary recruits who are more committed ideologically are better able to resolve the moral conflict associated with committing atrocities and are therefore less likely to exhibit symptoms of MI. Our findings do not lend credence to this hypothesis.

In sum, three forces seem to be at work in driving these complex interactions. As the dashed line is always above the solid line, the risk of MI for perpetrators who joined a faction as a teenager, regardless of their history of abduction, is higher. The estimated effect of voluntary recruitment is weaker than the effect of perpetration and applies only to non-perpetrators. Age of joining has the weakest association, as younger non-perpetrators who joined a faction voluntarily have a lower average risk of MI. The perpetration effect appears to be the strongest and dominates the other two. Younger non-abductees who committed no atrocities have a slight advantage because of their age. Witnesses of violence do not appear to have notably high predicted MI scores, with the score never exceeding 15 out of a possible 60 points.

**Fig. 2:**
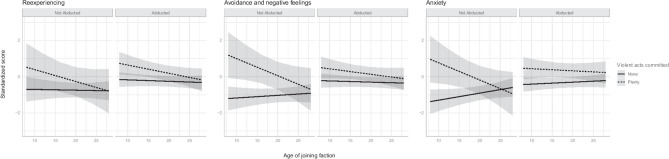
Moderation Effects of Age and Abduction History on Reexperiencing, Avoidance and Negative Feelings, and Anxiety

To gain further insights into the mechanism of MI development, the composite MI indicator was disaggregated into the three constituting clusters: (i) reexperiencing, (ii) avoidance and negative feelings, and (iii) anxiety. Their standardized scores were used as the dependent variables and a similar set of independent variables were used in three separate regression models. The results are summarized in Fig. 2. The patterns for avoidance and anxiety are similar to those shown in Fig. 1, although the relationship between perpetration and avoidance is not statistically significant for abductees. The relationship between perpetrating violent acts and reexperiencing is not statistically significant either. The positive slope of the solid line for non-perpetrators in Fig. 1 is almost flat in Fig. 2 (the reexperiencing panel), suggesting that the joining age effect can no longer be detected in the reexperiencing cluster. The volunteering effect is also weaker in the reexperiencing cluster. All these suggest that the perpetration effects on our MI indicator might be primarily driven by the avoidance and the anxiety clusters, and the volunteering effect is primarily driven by the avoidance and anxiety clusters as the solid lines are notably lower for young non-abductees in the corresponding graphs. The results related to the roles of avoidance, negative emotions and anxiety are consistent with the findings by Hoffman et al. (2018) and Jinkerson and Battles (2019). The role of reexperiencing is less evident in our sample of former child soldiers. This is contrast to the findings of a longitudinal study on MI among refugees (Nickerson et al., 2020).

## Conclusion

This study investigates the moderating effects of abduction and age on the relationship between perpetrating atrocities and the presence of MI symptoms in former child soldiers. Atrocities are positively associated with MI even when they were committed involuntarily. Younger former child soldiers in our sample appear to be more resilient to trauma stress than adults, provided that they committed no violent acts against other people. Our overall findings are also in line with the conceptual model of MI about the relevance of avoidance in MI.

Our analysis is subject to limitations. First, since participation in the reintegration program was voluntary, those with symptoms of psychological withdrawal were less likely to join the program. As withdrawal is a symptom of MI (Litz et al., 2009), and those who participated were likely to have milder symptoms of avoidance and withdrawal, the true effect of MI is likely to be greater than what is reported due to self-selection. Therefore, our estimates might be interpreted as a conservative, lower bound effect of perpetration on MI. Second, the aggregate indicator of MI is not based on existing psychometric scales such as the Moral Injury Events Scale (MIES; Nash et al., 2013) as the survey was conducted before the concept of MI became popularized. Although the aggregated indicator of MI symptoms used in the analysis covers many defining characteristics of MI relevant to former child soldiers and it captures effects of perpetration after controlling for life-threatening and traumatic events, the regression results should be treated as a preliminary test to the hypotheses. Finally, the analysis uses the age of joining a faction as a proxy for the age of committing atrocities. Though likely to be coincided (Thompson, 1999; Wessells, 1997), the two are not necessarily the same. Therefore, the estimation is likely to underestimate the impact of the perpetrations because they were likely to be committed after joining a faction. Overall, due to the potential impacts of MI on the mental wellbeing of ex-combatants, policy makers should consider mainstreaming MI-specific interventions in future DDRR programs for former child soldiers.
